# Apoptotic action of botulinum toxin on masseter muscle in rats: early and late changes in the expression of molecular markers

**DOI:** 10.1186/s40064-016-2680-9

**Published:** 2016-07-07

**Authors:** Young-Min Moon, Min-Keun Kim, Seong-Gon Kim, Tae-Woo Kim

**Affiliations:** Department of Orthodontics, School of Dentistry, Dental Research Institute, Seoul National University, Seoul, Korea; Department of Oral and Maxillofacial Surgery, College of Dentistry, Gangneung-Wonju National University, 7 Jukhyun-gil, Gangneung, 210-702 Korea

**Keywords:** Botulinum toxin-A, p65, Bcl-2, Type II myosin, TUNEL, Apoptosis, Masseter muscle

## Abstract

The purpose of this study was to compare the early or late expression levels of p65, Bcl-2, and type II myosin and the frequency of TUNEL-positive nuclei in the rat masseter muscle after injection of different concentrations of botulinum toxin-A (BTX-A). We injected either 5 U or 10 U of BTX-A into both masseter muscles of the rat. As a control group, the same volume of saline was injected. After 2 or 12 weeks, the animals were sacrificed. Subsequently, a biopsy and immunohistochemical staining of the samples were performed using a p65, Bcl-2, or type II myosin antibody. Additionally, a TUNEL assay and transmission electron microscopic analysis were performed. The expression of p65, Bcl-2, and type II myosin increased significantly with increasing concentrations of BTX-A at 2 weeks after BTX-A injection (*P* < 0.05). The number of TUNEL-positive nuclei was also significantly increased in the BTX-A-treated groups in comparison to the saline-treated control at 2 weeks after BTX-A injection (*P* < 0.05). Elevated expression of Bcl-2 was also observed in 10-unit BTX-A-treated group at 12 weeks after injection (*P* < 0.05). At 12 weeks after injection, the number of enlarged mitochondria was increased, and many mitochondria displayed aberrations in cristae morphology after BTX-A injection. In conclusion, BTX-A injection into the masseter muscle increased the expression level of p65, Bcl-2, and type II myosin at an early stage. The morphological changes of mitochondria were more evident at 12 weeks after injection.

## Background

Botulinum toxin-A (BTX-A) is a protein produced by bacterium *Clostridium botulinum* (Schiavo et al. 1992). Synaptosomal-associated protein of 25 kDa (SNAP-25) has been regarded as the only target for BTX-A until recently (Rossetto et al. 2014). BTX-A mediated SNAP-25 proteolysis inhibits acetylcholine release from nerve endings (Rossetto et al. 2014). Since the approval of its usage by the US Food and Drug Administration (FDA) in 1989, BTX-A has been widely used in the field of oral and maxillofacial surgery for the treatment of temporomandibular disorder (Kim et al. 2016) and masseteric hypertrophy (von Lindern et al. 2001). The therapeutic dosage of BTX-A has been regarded as safe with few complications (Mahant et al. 2000). However, small amounts of BTX-A may enter into circulation, and its duration is not yet determined (Carruthers and Carruthers 1997).

Recently, new findings suggest that BTX-A may have antimitotic and antitumor properties (Matak and Lackovic 2015). BTX-A reduces fibroblast proliferation originating from keloid scarring (Zhibo and Miaobo 2008). BTX-A induces apoptosis mediated by capase-3 and capase-7 in breast cancer cells (Bandala et al. 2013). Interestingly, these fibroblasts and breast cancer cells do not express SNAP-25 (Matak and Lackovic 2015). BTX-A also activates apoptotic pathways in the prostate via sympathetic nerve impairment (Gorgal et al. 2012). Positive deoxynucleotidyl transferase biotin dUTP nick-end labelling (TUNEL) staining is increased in rat and dog prostates following BTX-A injection (Doggweiler et al. 1998; Chuang et al. 2006). As BTX-A is widely used for the correction of strabismus, the potential for apoptosis to occur in the extraocular muscle following BTX-A injection has also been studied (Croes et al. 2007). The proliferation zone of the mandibular condyle shows increased apoptosis following BTX-A injection (Kim et al. 2008). Although clinical application of BTX-A has increased, potential induction of apoptosis in the masseter muscle has not been studied.

Endoplasmic reticulum (ER) stress-induced inflammation is mainly mediated by Jun NH2-terminal kinase (JNK) (Zhang and Kaufman 2008a) and nuclear factor-kappa B (NF-κB) (Hu et al. 2006; Zhang and Kaufman 2008a). Ultimately, uncontrolled and excessive ER stress will lead to apoptosis and cell death (Ron and Walter 2007; Zhang and Kaufman 2008b). The Bcl-2 family members are involved in ER stress-induced apoptosis (Logue et al. 2013) to the extent that apoptotic signaling can be modulated by regulating Bcl-2 level (Gomez et al. 2007; Kurata et al. 2011). In addition, overexpression of Bcl-2 reduces the loss of mitochondrial membrane potential and protects cells against ER stress-induced apoptosis (Heath-Engel et al. 2008). Therefore, measuring NF-κB and Bcl-2 level would be useful indicators for cellular apoptosis. The p65 is a family of NF-κB and the expression of p65 is increased by exercise-induced muscle trauma (Crane et al. 2012). In addition, the dynamic shifts in mitochondrial morphology coincide with a number of physiological events, such as transitions between different respiratory states and cristae remodeling during apoptosis (Parra et al. 2011).

The composition of myofiber-type is changed by oxidative damage (Koutakis et al. 2014). Peripheral artery disease produces myopathy and myosin type II (MYO2) and MYO1/2 mixed fiber are increased (Koutakis et al. 2014). Short-term limb immobilization induces decreased MYO1 and MYO2a area (Yasuda et al. 1985). BTX-A injection induces increased expression of gene related to impaired mitochondrial biogenesis at 1 week (Mukund et al. 2014). BTX-A injection to muscles increases the expression of proteins related to cellular stress response (Han et al. 2013). As BTX-A increases stress-response to injected muscle, composition of MYO type will be changed after BTX-A injection. Actually, MYO2a composition is increased in the masseter muscle of pig after BTX-A injection (Gedrange et al. 2013). Therefore, MYO2a could be used as an indicator of the stress induced by BTX-A injection.

Serious complications such as death and seizures are reported after BTX-A injection, and these complications are associated with high-dose application of BTX-A and underlying systemic disease (Coté et al. 2005). The purpose of this study was to compare the expression levels of MYO2a, p65 and Bcl-2 and the frequency of TUNEL-positive nuclei in the rat masseter muscle after injection of BTX-A at different concentrations. Additionally, the ultrastructure of masseter muscle was studied using transmission electron microscopic (TEM) images.

## Methods

### Animals and experimental design

Male Wistar rats aged 18 weeks were purchased from Samtako (Seoul, Korea). They were housed individually at a controlled temperature (20–22 °C) and hygrometry (approximately 40 %) in a 12 h light: 12 h darkness cycle. They had free access to water. During the adaptation period (first week) all rats were fed ad libitum with a control semi-synthetic diet (4 % lipids from soya vegetal oil, 74 % carbohydrates from sucrose and cornstarch, and 14 % proteins from casein, supplemented with standard vitamins and mineral mix), following classical recommendations. All diets were prepared within Gangneung-Wonju National University facilities. All groups were maintained ad libitum for 7 days receiving a diet similar to the adaptation diet while daily spontaneous intake was measured (26.1 ± 4.1 g/d, n = 30). At the end of the normal diet period, rats (20 weeks-old) were separated: the control group received a saline injection into both masseter muscles (group 1, n = 10), and the others were separated into two groups for the BTX injection study (n = 10 per group). All re-feeding diets were the same as the ad libitum control period. To measure food intake all groups were individually housed. Group 1 was the saline-injected group. Animals in group 2 received a 5-unit BTX-injection to each masseter muscle, and group 3 received a 10-unit BTX-injection to each masseter muscle. Half of the animals were sacrificed at 14 days after the injection. The other animals were sacrificed at 12 weeks after injection. All procedures were conducted according to the guidelines of laboratory animal care and were approved by the Gangneung-Wonju National University for animal research (GWNUA-2015-35).

### TUNEL assay and immunohistochemical determination of p65 and Bcl-2 in rat masseter muscle

The samples were harvested, decalcified in 5 % nitric acid for 5 days, and dehydrated in ethyl alcohol and xylene. After separation of the calvarial bones, the head samples were embedded in paraffin blocks. The paraffin blocks were sliced into sections. The section with the occlusal plane area was selected. The TUNEL assay was performed using the DeadEnd™ fluorometric TUNEL system (Promega, Madison, WI, USA). The detailed procedure was consistent with the manufacturer’s protocol. The number of TUNEL positive nuclei in 10 random fields at ×400 magnification in the masseter muscle was evaluated by computer-assisted image analysis. DAPI stain was used as a counterstaining.

To determine the level of expression of MYO2a, p65, and Bcl-2, immunohistochemical staining was performed using anti-MYO2a antibody (sc-71632: Santa Cruz Biotech, Santa Cruz, CA, USA), anti-p65 antibody (sc-8008: Santa Cruz Biotech), and anti-Bcl-2 antibody (sc-492: Santa Cruz). Paraffin-embedded tissues from rat masseter muscles were prepared. For antigen retrieval, sections were incubated in trypsin for 7 min at 37 °C. The primary antibody dilutions were as follows: p65 and BCL2 for 1:50. The immunohistochemical procedures were performed as described in a previous publication. The negative controls were sections stained without primary antibodies.

Stained sections were examined in an Olympus BX51 (Olympus, Tokyo, Japan) microscope. Digital images of the selected sections were captured with a digital camera (DP-73; Olympus, Tokyo, Japan). The images were analyzed using Sigma Scan pro (SPSS, Chicago, IL). To quantify the immunohistochemical reaction intensity, the positive intensity immuno-staining in 10 random fields at ×100 magnification in the masseter muscle was evaluated by computer-assisted image analysis after image transformation to grayscale. The staining intensity was expressed as the mean intensity value (0: lowest intensity, 255: highest intensity). The samples were not counterstained so that the absorbance would be solely attributable to the product of the immunohistochemical reaction.

### Transmission electron microscopic (TEM) analysis

The specimen preparation was referred to Cheongwon Center, KSBI. The detailed procedure was as follows. The masseter muscles were cut as 1 mm × 1 mm × 1 mm. These specimens were put into 2.5 % glutaraldehyde in 1 M PB buffer overnight. The specimens were washed with 0.1 M PB buffer for 10 min three times. After removing supernatant solution, they were put into 1 % OsO4 for 1 h. The specimens were washed with 0.1 M PB buffer for 10 min three times, again. The specimens were washed with distilled water for 5 min two times. Then, the specimens underwent a dehydration process with a graded series of ethanol. The specimens were substituted with 100 % propylene oxide for 30 min two times. Then, the specimens were embedded in propylene oxide and Epon812 media. The embedded specimens were cut as ultra-thin Sections (70 nm thickness) using Ultra-Microtome (ULTRACUT UCT, LEICA, installed at the Korea Basic Science Institute). The prepared section was referred to Gangneung Center, KSBI for taking a TEM image. The cut section was placed on a 150 mesh grid. The specimens were observed with JEM-2100F (JEOL, Japan, installed at Korea Basic Science Institute) under 200 keV.

### Statistical analysis

SPSS for Windows ver. 19 (IBM Co., Armonk, NY, USA) was used for statistical analysis. The differences among groups were evaluated by ANOVA. For post hoc tests, Bonferroni’s method was used. The statistical significance level was set at *P* < 0.05.

## Results

**Fig. 1:**
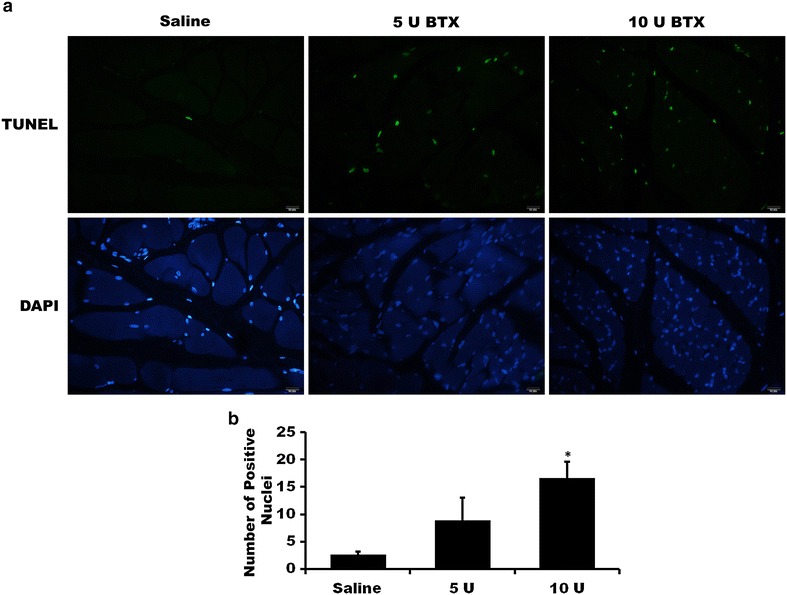
The results of the TUNEL assay at 2 weeks after injection. **a** The number of highly fluorescent nuclei in the masseter muscle was increased in the 5 U and 10 U BTX-A-treated groups in comparison to the saline-treated control group (original magnification ×400). DAPI staining was used as a counterstaining. **b** The TUNEL assay demonstrated that the number of TUNEL-positive nuclei was significantly higher in the 10 U BTX-A-treated group than in the saline-treated group (*P < 0.001)

The results of the TUNEL assay demonstrated that the number of highly fluorescent nuclei in the masseter muscle was increased in the 5 U and 10 U BTX-A-treated groups in comparison to the saline-treated control group at 2 weeks after injection (Fig. 1a). The TUNEL assay demonstrated that the number of TUNEL-positive nuclei was significantly higher in the 10 U BTX-A-treated group than in the saline-treated group (Fig. 1b; *P* < 0.001). The mean numbers of TUNEL-positive nuclei were 2.60 ± 0.55, 8.80 ± 4.21, and 16.60 ± 2.97 for the saline, 5 U, and 10 U BTX-A treatments, respectively. The post hoc test revealed differences between the groups treated with 10 U BTX-A and the other groups, with significantly higher values in the 10 U BTX-A group than in the saline-treated control and 5 U BTX-A-treated group (*P* < 0.001 and 0.004 for the saline-treated group and the 5 U BTX-A-treated group, respectively). However, there was no difference in the number of TUNEL-positive nuclei among groups at 12 weeks after injection (data not shown).

**Fig. 2:**
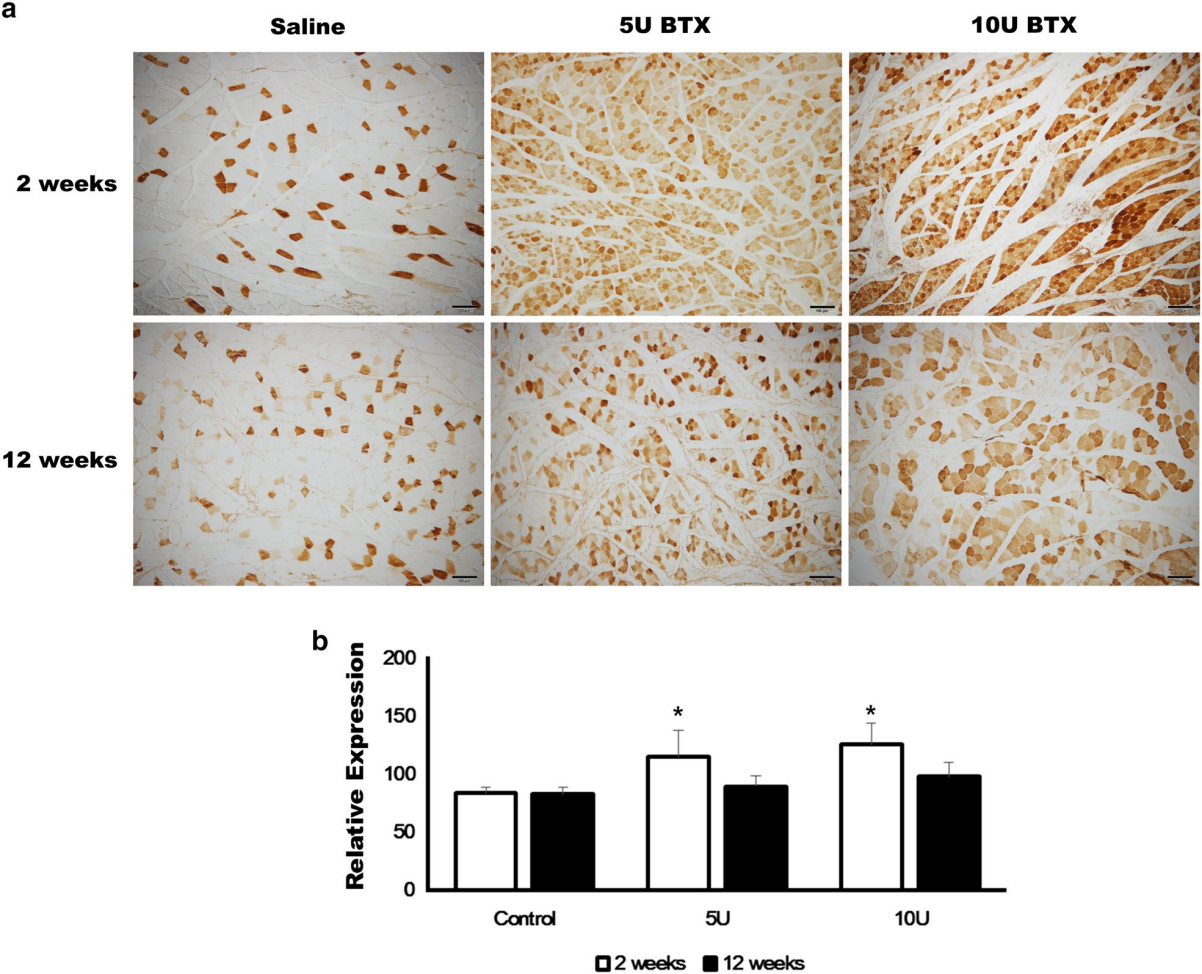
Immunohistochemical findings of MYO2a expression. **a** Both BTX-A-treated groups displayed higher MYO2a expression than the saline-treated group (original magnification ×100). **b** The 5 U BTX-A-treated group and 10 U BTX-A-treated group had significantly higher values compared with the saline-treated group at 2 weeks after injection (*P < 0.05)

The immunohistochemical findings demonstrated that there was a statistically significant difference among groups in MYO2a expression at 2 weeks after injection. Both BTX-A-treated groups displayed higher MYO2a expression than the saline-treated group (Fig. 2a, b). The expression level of MYO2a was 83.61 ± 4.90, 115.07 ± 22.77, and 125.57 ± 18.39 in the saline-treated, 5 U BTX-treated-, and 10 U BTX-treated groups, respectively, at 2 weeks after injection. The difference among groups was statistically significantly (P = 0.006). In the post hoc test, the 5 U BTX-A-treated group and 10 U BTX-A-treated group resulted in significantly higher values compared with the saline-treated group (P = 0.040 and 0.007, respectively). However, there was no statistically significant difference among groups in MYO2a expression at 12 weeks after injection (P > 0.05). The expression level of MYO2a was 82.79 ± 5.73, 89.57 ± 9.14, and 98.25 ± 11.82 in saline-treated, 5 U BTX-treated, and 10 U BTX-treated groups, respectively, at 12 weeks after injection. Both BTX-A-treated groups displayed a similar level of MYO2a expression to the saline-treated group.

**Fig. 3:**
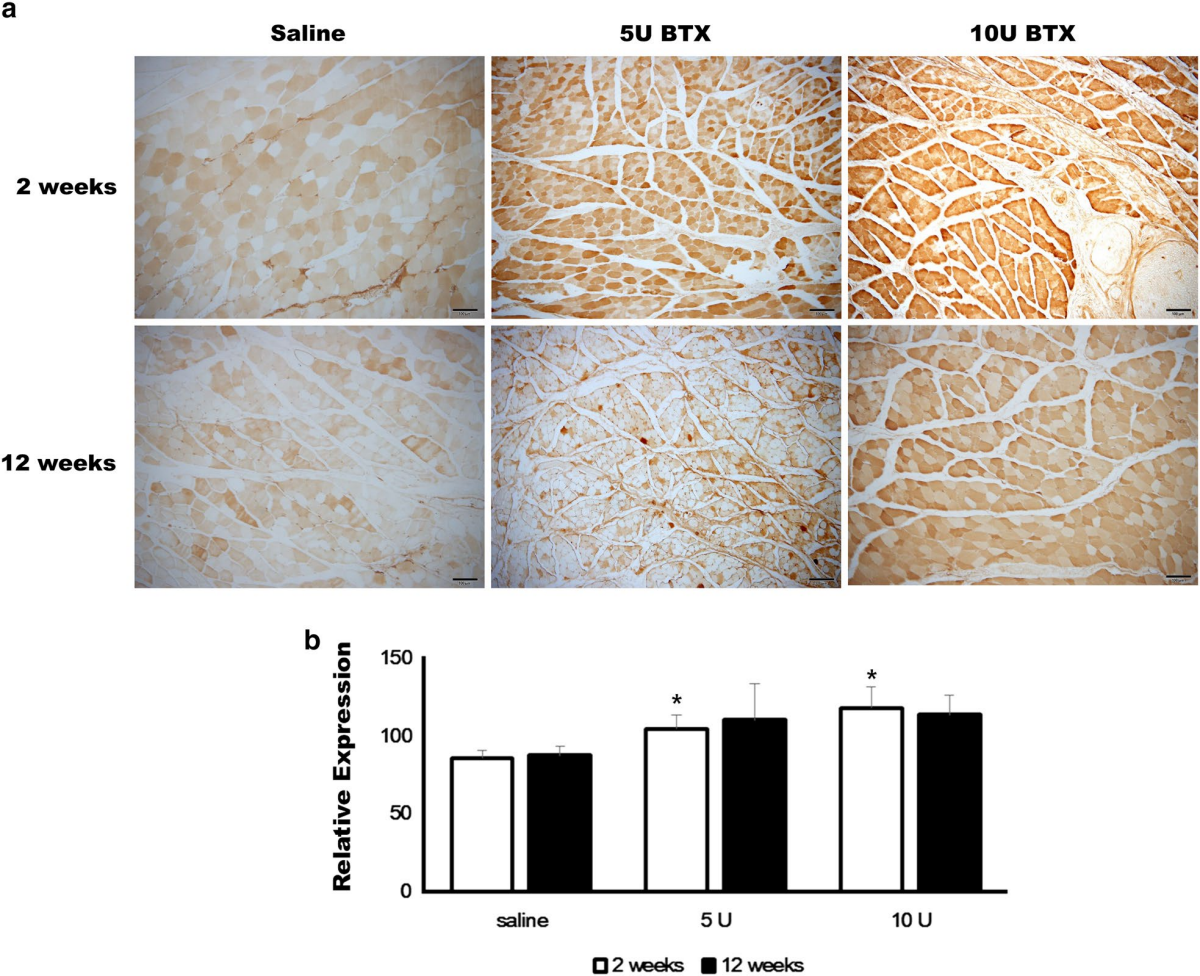
Immunohistochemical findings of p65 expression. **a** The expression of p65 was increased in both BTX-treated groups at 2 weeks after injection (original magnification ×100). **b** The 5 U BTX-A-treated group and 10 U BTX-A-treated group had significantly higher values compared with the saline-treated group at 2 weeks after injection (*P < 0.05)

The expression of p65 was significantly different among groups at 2 weeks after injection (Fig. 3, P = 0.001). The relative expression of p65 was increased in both BTX-treated groups at 2 weeks after injection. The expression level of p65 was 85.94 ± 4.65, 104.55 ± 8.65, and 117.94 ± 13.79 in the saline-treated, 5 U BTX-treated, and 10 U BTX-treated groups, respectively, at 2 weeks after injection. In the post hoc test, 5 U and 10 U BTX-A treatment resulted in significantly higher values compared with the saline-treated group (P = 0.033 and 0.001, respectively). The elevated p65 expression was maintained at 12 weeks after injection (Fig. 3). The expression level of p65 was 87.74 ± 5.61, 110.31 ± 23.08, and 113.56 ± 12.41 in saline-treated, 5 U BTX-treated, and 10 U BTX-treated groups, respectively, at 12 weeks after injection. However, the difference among groups was not statistically significant (P > 0.05).

**Fig. 4:**
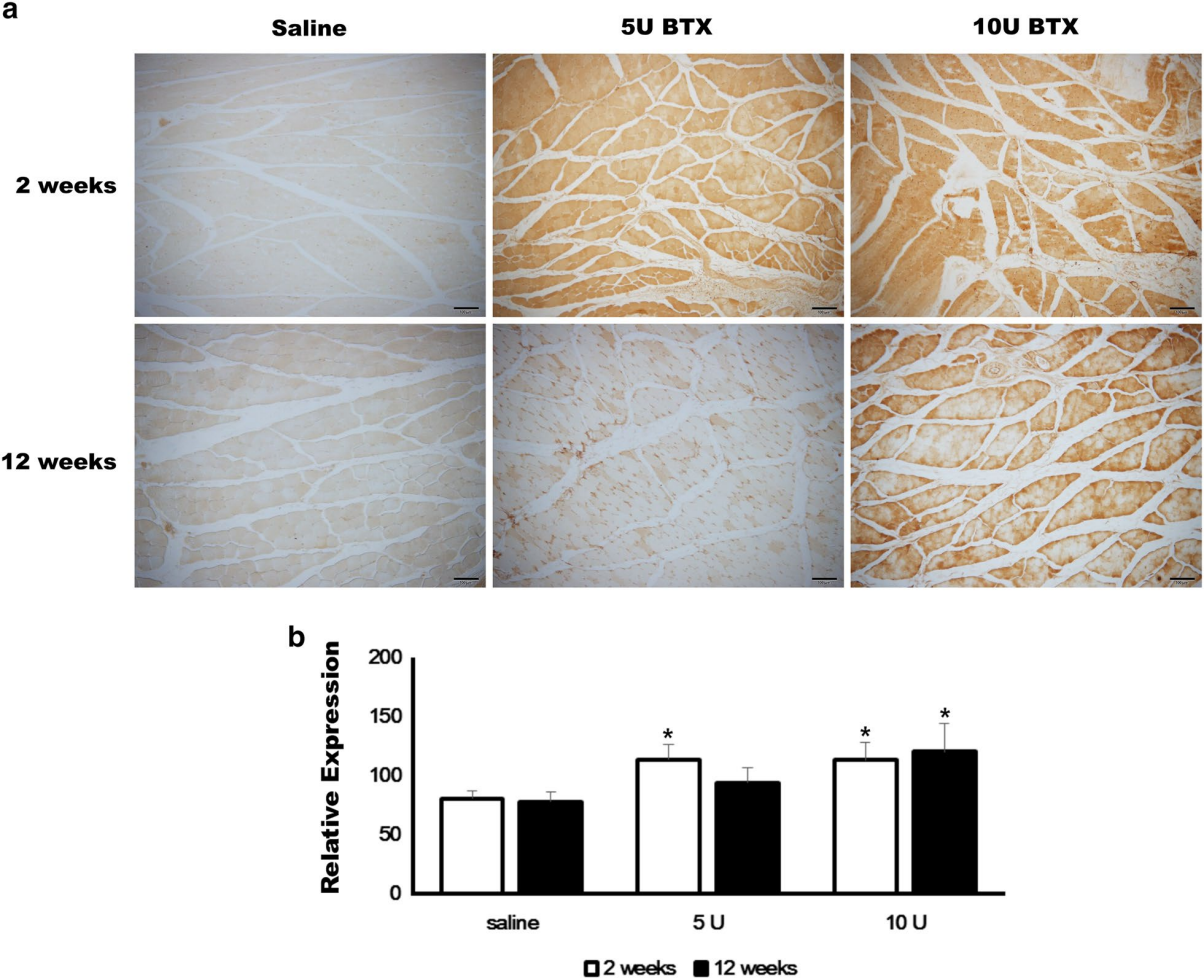
Immunohistochemical findings of Bcl-2 expression. **a** The expression of Bcl-2 was increased in both BTX-treated groups at 2 weeks after injection. The elevated Bcl-2 expression in the 10 U BTX-A-treated group was maintained at 12 weeks after injection (original magnification ×100). **b** The 5 U BTX-A-treated group and 10 U BTX-A-treated group had significantly higher values compared with the saline-treated group at 2 weeks after injection (*P < 0.05). The 10 U BTX-A-treated group had significantly higher values compared with the saline-treated group at 12 weeks after injection (*P < 0.05)

The expression of Bcl-2 was significantly different among groups at 2 weeks after injection (Fig. 4, P = 0.001). The relative expression of Bcl-2 was increased in both BTX-treated groups at 2 weeks after injection. The expression level of Bcl-2 was 80.65 ± 6.07, 113.45 ± 12.75, and 113.08 ± 15.03 in saline-treated, 5 U BTX-treated, and 10 U BTX-treated groups, respectively, at 2 weeks after injection. In the post hoc test, 5 U and 10 U BTX-A treatment resulted in significantly higher values compared with the saline-treated group (P = 0.003 for both groups). The elevated Bcl-2 expression was maintained at 12 weeks after injection (Fig. 4). The expression level of Bcl-2 was 78.20 ± 8.26, 93.92 ± 12.40, and 120.30 ± 23.56 in saline-treated, 5 U BTX-treated, and 10 U BTX-treated groups, respectively, at 12 weeks after injection. The difference among groups was statistically significant (P = 0.005). In the post hoc test, the 10 U BTX-A-treated group had significantly higher values compared with the saline-treated group (P = 0.004).

**Fig. 5:**
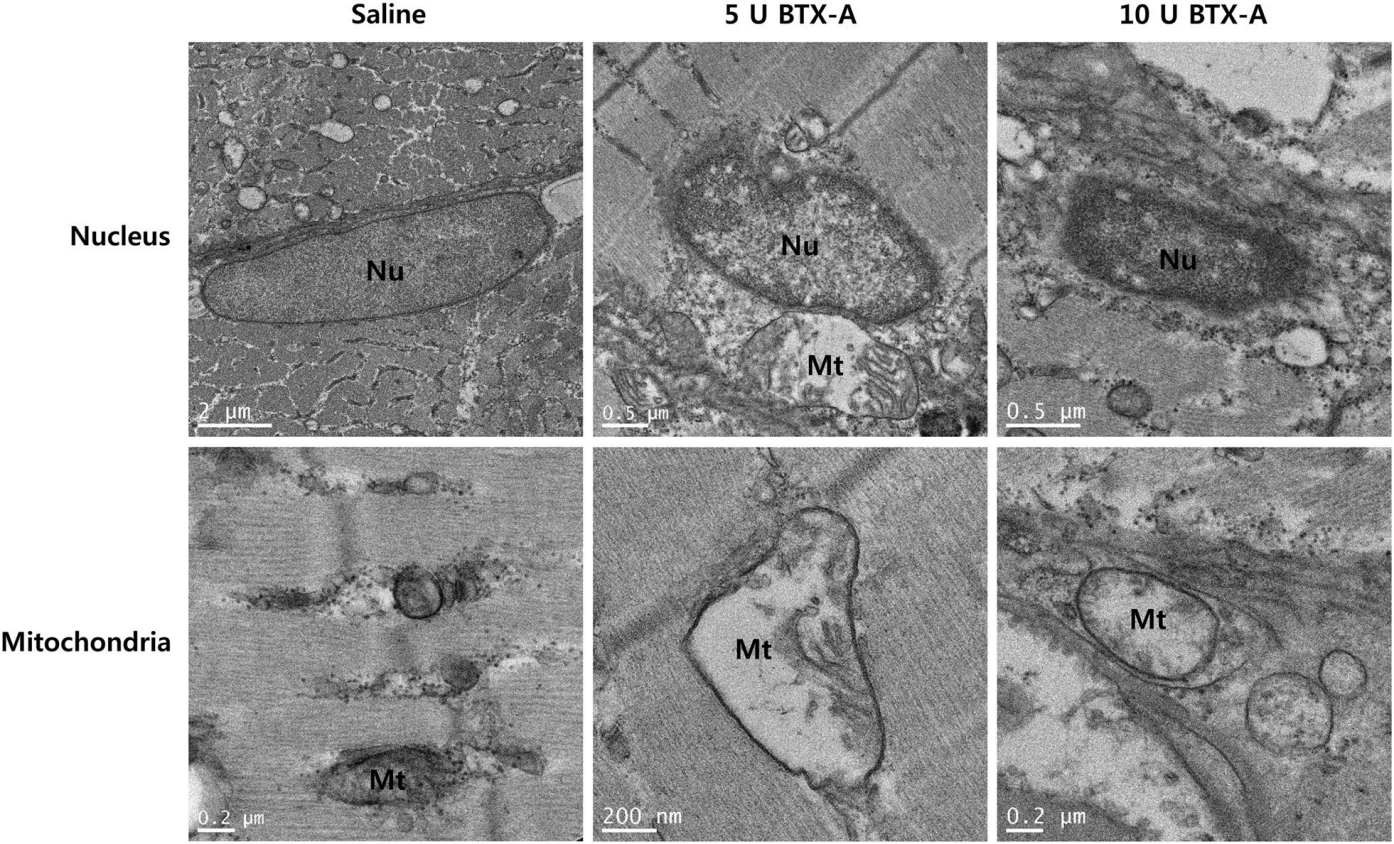
Analysis of TEM of muscular tissue 2 weeks after BTX-A or saline injection. Shrunken nuclei with chromatin condensation (Nu) was evident in both BTX-A-treated groups. Swollen mitochondria (Mt) with aberrant cristae were sporadically found. Mitochondria with aberrant cristae (Mt) in the 10 U BTX-A-treated group was encompassed by membranes and underwent autophagy formation

**Fig. 6:**
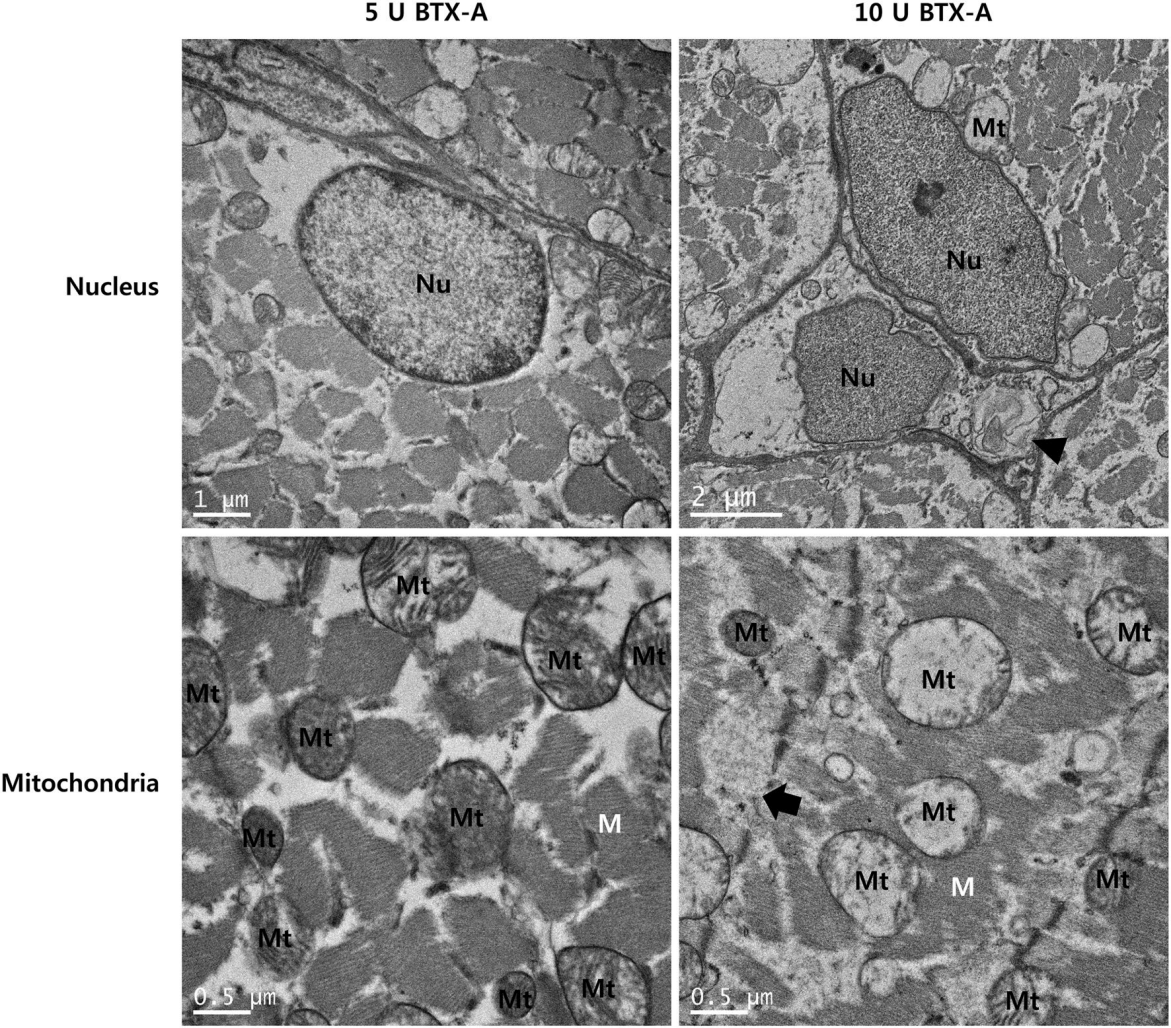
Analysis of TEM of muscular tissue 3 months after BTX-A injection. Nuclei (Nu) had intact membranes in both groups. Mitochondria (Mt) having aberrant cristae contacted the nucleus in the 10 U BTX-A group. Myelinic figure (*arrow head*) compatible with a toxic myopathy was found. Enlarged mitochondria were found in both groups. However, the outer membrane of mitochondria was intact in most mitochondria, though they had aberrant cristae. Mitochondria having aberrant cristae were more common in the 10 U BTX-A-treated group than in the other groups. In addition, abruption of Z-line (*arrow*) was observed in the 10 U BTX-A-treated group

Considering the profound myofilaments abnormalities observed in the masseter muscle of BTX-A-treated rats, the morphology of mitochondria in the muscle fibers was compared in TEM images. Destruction of myofibrils was not prominent at 2 weeks following 10 U BTX-A treatment (Fig. 5). However, destruction of mitochondrial structure and subsequent autophagy formation were observed at 2 weeks after BTX-A injection. At 12 weeks after BTX-A injection, muscle fibers had numerous large mitochondria with loosely packed cristae in the 5 U BTX-A group (Fig. 6). These mitochondria were interspersed between myofibrils. In addition, regional destruction of myofibrils was also found. This type of myofibril destruction was more extensive in the 10 U BTX-A group at 12 weeks after injection. Mitochondrial degeneration and myelinic figure was noticed. Many mitochondria showed aberrations in cristae morphology, and the Z line was discontinuous.

## Discussion

In this study, the relative expressions of MYO2a, p65, and Bcl-2 were increased 2 weeks after BTX-A injection. In addition, regional chromatin condensation and ruptured mitochondrial membranes were observed with increased number of TUNEL positive nuclei. Elevated levels of MYO2a, p65, and Bcl-2 were generally decreased 12 weeks after BTX-A injection. However, enlarged mitochondria and aberrations in cristae morphology were evident in the 10 U BTX-A treatment group. Increasing apoptotic stress at 2 weeks after BTX-A injection might be a direct effect of the toxin on the muscle cells. However, enlarged mitochondria and aberrations in cristae morphology in the 10 U BTX-A treatment group at 12 weeks after injection might be due to an imbalance between physical loading and non-paralyzed muscle cells.

The composition of type II fibers is known to be increased after BTX-A injection into the masseter muscle (Moon et al. 2015). Our study also demonstrated elevated levels of MYO2a at 2 weeks after BTX-A injection. The composition of type II fibers increases following heavy resistance loading to skeletal muscles (D’Antona et al. 2006). As BTX-A dose-dependently denervated the masseter muscle, it is expected that the number of paralyzed muscle cells would increase dose-dependently. Accordingly, the loading to residual functional fibers would be increased. Interestingly, decreased food intake rapidly recovered following BTX-A injection in both rats (Moon et al. 2015) and humans (Freund et al. 1999). As small functional fibers received heavy load, this should induce changes in metabolism. Aberrations in mitochondrial cristae morphology are accompanied by changes in metabolism (Parra et al. 2011; Nasrallah and Horvath 2014), and together with the down-regulation of optic atrophy protein-1 or mitofusin, these aberrations trigger alterations of the inner mitochondrial membrane structures, which leads to fragmented mitochondria with greatly reduced oxygen consumption and electrochemical potential (Chen and Chan 2005). BTX-A irreversibly binds to the motor nerve endings within 90 min (Klein 2003). Consequently, the paralysis of the masseter muscle is evident immediately after BTX-A injection (Park et al. 2015). As the effect of BTX-A injection lasts more than 3 months (Ma et al. 2004), the stress from the metabolic imbalance will accumulate until 3 months after injection. Subsequently, the size of mitochondria is enlarged due to the demands of metabolic requirements.

Some mitochondria showed ruptured outer membranes and destruction of cristae morphology at 2 weeks after BTX-A injection. Damaged mitochondria were encompassed by membranes and underwent autophagy formation. Some toxin actions of BTX-A might be independent of SNAP-25 (Matak and Lacković 2015). BTX-A increases the apoptotic index in human prostate cancer cell lines that express neither SNAP-25 transcript nor protein (Karsenty et al. 2009; Matak and Lacković 2015). The mitochondria in the extraocular muscles show significant changes following BTX-A injection in the acute phase (Spencer and McNeer 1987). The number of TUNEL-positive nuclei was increased, and many nuclei showed condensed chromatin at 2 weeks after BTX-A injection. BTX-A application reduces cellular proliferation in the fibroblasts originating from hypertrophic scars (Zhibo and Miaobo 2008). Uncontrolled and excessive ER stress will lead to apoptosis and cell death (Ron and Walter 2007; Zhang and Kaufman 2008b). However, increased TUNEL positive nuclei and autophagy formation might not result in complete destruction of muscle fibers. As the muscle cell is a multi-nucleated cell, denervation-induced apoptosis eliminates individual nuclei without destroying the entire fiber (Tsai et al. 2010). Autophagy formation also increases lifespan of affected cells (Lee et al. 2006).

ER stress-induced inflammation is mainly mediated by nuclear factor-kappa B (Hu et al. 2006; Zhang and Kaufman 2008a). As the expression level of p65 was increased by BTX-A injection in this study, ER stress-induced inflammation was evident in the BTX-A injected masseter muscle. Accordingly, overexpression of Bcl-2 is important in the protection of cells against ER stress-induced apoptosis (Heath-Engel et al. 2008). The proapoptotic and anti-tumor activity of BTX-A might not be related to SNAP-25 (Matak and Lackovic 2015). The light chains of BTX-A are mainly localized to the plasma membrane in non-neuronal cell lines (Fernández-Salas et al. 2004) and might impair mitochondrial respiration. This mechanism might also be related to the changes following BTX-A injection in the acute phase. The exact mechanism has yet to be elucidated.

Immobility of the skeletal muscle, such as BTX-A mediated paralysis, triggers the unfolded protein response and increases subsequent ER stress (Alibegovic et al. 2010). ER stress can accumulate in the non-pathological skeletal muscle (Deldicque et al. 2012). Aberrations in mitochondrial cristae morphology and mitochondrial enlargement were evident at 12 weeks after BTX-A injection. However, most mitochondria had intact outer membranes. Bcl-2 family members have a pivotal role in the control of mitochondrial apoptosis (Shariat et al. 2005). Bcl-2 and Bcl-xL protect the cell from apoptosis, but Bax induces apoptosis (Shariat et al. 2005). Interestingly, the level of Bcl-2 expression was still significantly increased at 12 weeks after 10 U BTX-A injection. The overexpression of Bcl-2 can delay mitochondrial apoptosis (Shariat et al. 2005). Therefore, the intact mitochondrial outer membrane might be due to the protective function of Bcl-2. In neurectomy, the expression of cytochrome c, which is released when rupturing the mitochondrial outer membrane, is decreased (Davatz et al. 2007).

## Conclusion

Collectively, BTX-A injection induced immobilization of the masseter muscle seemed to increase apoptotic stress in the masseter muscle via ER stress-induced inflammation. Overexpression of Bcl-2 might protect muscle cells from BTX-A induced apoptotic stress.
